# Giant Keratocystic Odontogenic Tumor: Three Cases and Literature Review

**Published:** 2013-10

**Authors:** Alexandre Caixeta Guimarães, Mariana Dutra de Cassia Ferreira Santos, Guilherme Machado de Carvalho, Carlos Takahiro Chone, Leopoldo Nizam Pfeilsticker

**Affiliations:** 1 *Department of Otorhinolaryngology, University of Campinas (UNICAMP), School of Medical Sciences (FCM), São Paulo, Brazil.*

**Keywords:** Keratocystic tumor, Jaw, Mandible, Odontogenic tumor

## Abstract

**Introduction:**

A keratocystic odontogenic tumor is a benign intra-bone mass originating from dental lamina or its residue. It represents 2–11% of jaw cysts, and has a slow but aggressive growth. The evaluation of molecular characteristics, immunohistochemistry, and genetic expression currently have no established classification regarding the evolution and pathophysiologic pattern of these lesions.

**Case Report:**

This is a clinical retrospective study with a full analysis of patient history regarding physical evaluation, radiologic images, pathology results, and surgical resection. We performed a major literature review concerning current concepts relating to its biological characterization. Three cases of keratocystic odontogenic tumor were identified. Two of the cases were large, with aggressive behavior and significant bone destruction and recurrence, which had been overlooked for more than a decade. The third case had an early diagnosis, and the treatment led to full recovery and complete healing.

**Conclusion:**

The keratocystic odontogenic tumor is a benign lesion with slow growth, which lends itself to a more conservative treatment, even in cases of large lesions. A better understanding of these tumors, both at the biological and molecular level, could lead to guidelines for treatment and prognosis of such patients.

## Introduction

Odontogenic tumors are considered rare neoplasms, with a challenging diagnosis and treatment. The numerous publications concerning these tumors tend to be case reports with unusual histopathologic or clinical behavior. Furthermore, publications prior to 2006 were based on the 1971 World Health Organization (WHO) histological classification. 

In 2005, the new histological classification of odontogenic tumors by the WHO reclassified the odontogenic keratocyst as benign intra-osseous neoplasia, calling it a keratocystic odontogenic tumor (KOT) (1). Originating from the dental blade or from its residue, this tumor affects the bearing areas of the teeth (2), and represents 2–11% of all mandibular cysts. Occurring at any age, these tumors are more common in men than women, at an approximately 2:1 ratio, and are very aggressive locally, with recurrence rates ranging from 3–60% (3). Although some KOT characteristics are considered typical of neoplasias, particularly the high proliferation rate of the epithelial cells, its behavior and treatment remain controversial (4).

Recent molecular and genetic investigations targeted towards odontogenic tumors, especially the KOT, suggest its biological origins, thus broadening the understanding of its pathophysiology (1). Although the recurrence of prognostic factors based on clinical pathological and immunohisto- chemical features remain undetermined; their use may become an important assessment of this neoplasia behavior, and may, eventually, define a personalized treatment approach (5). 

We describe three KOT cases, including two large cases with aggressive behavior, and review the current knowledge regarding their biological characterization.

## Materials and Methods

This is a retrospective clinical study, which evaluated three patients at the Maxillo-Facial Surgery Service from the Department of Otorhinolaryngology Head and Neck at the University of Campinas Teaching Hospital (Campinas, Sao Paulo, Brazil) during 2011. 

The work consisted of a complete review of medical records from patients who underwent surgical treatment for mandibular lesions with a final diagnosis of KOT, in addition to a literature review regarding its biological characterization.

All patients underwent preoperative 3D reconstruction computed tomography (CT) scans followed by surgeries, performed by the same team, with histopathological diagnostic confirmation. They were followed postoperatively with clinical and radiographic control. Two cases presented large and aggressive behavior lesions, with distinct evolutions. 

A medical literature review was performed using PubMed/ MedLine, without research limits, with the MesH terms: keratocystic tumor; mandible; odontogenic tumor, immunohistochemistry. This study followed the institution Ethics Committee guidelines.

***Case 1***

A female patient, 53 years of age, with a 1-year history of bulging in the left ramus of the mandible, noticed after dental treatment, without any pain, bleeding, limited mandibular movement or weight loss complaints. The patient had no other history of disease. 

During a physical examination an enlargement was noticed in the left ramus of the mandible region, approximately 8 cm wide, painless, without involving the mouth floor, the gingivolabial sulcus, teeth, or cervical lymph nodes. 

A CT scan showed an insufflated lytic formation in the mandibular left ramus and angle, approximately 7 cm wide with the presence of thinness and rupture of the medial cortical with hypodense content and related dental elements preserved. The patient underwent surgical resection with complete removal of the intraoral cyst with thickened capsule filled with cornea formations. Histological analysis confirmed the KOT diagnosis. The left inferior alveolar nerve was identified and preserved. No grafts were used and complete regeneration of the surgical cavity was observed 4 years after the procedure.

***Case 2***

A female patient, 28 years of age, with complaints of bulging, pain, and hyperemia in the right mandibular angle region for 18 years and pus drainage recurrence in the oral cavity, always with spontaneous resolution. The lesion evolved with increasingly frequent relapses, bulging progression, worsening pain, difficulty in opening the mouth, and episodes of hypoesthesia in the right inferior alveolar nerve territory. No fever, weight loss or other complaints were reported. During a physical examination we observed bulging of approximately 10 cm in the body region and angle of the right mandible which was firm, painless, and without signs of inflammation. The buccal opening decreased by an average of 2.5 cm. Neck palpation was without alterations.

A CT scan showed expansive formation of approximately 5 cm in the ramus and the right angle region of the mandible with well-defined bone limits which was insufflated with hypo-attenuation and medial cortical rupture in the 3D reconstructions. The cuts without reconstruction presented no trabeculations or calcifications. There were no remaining teeth in the lesion. 

The lesion excision was performed intraorally, and was histologically diagnosed as a KOT; thus, confirming widespread erosion of the mandibular cortical bone corresponding to the lateral and medial cyst wall. Complete removal of the tumor was impossible to ensure in these sites and the bipolar cauterization of soft tissues was liberally used. The inferior alveolar nerve was not identified, and no cavity filling was used.

**Fig1:**
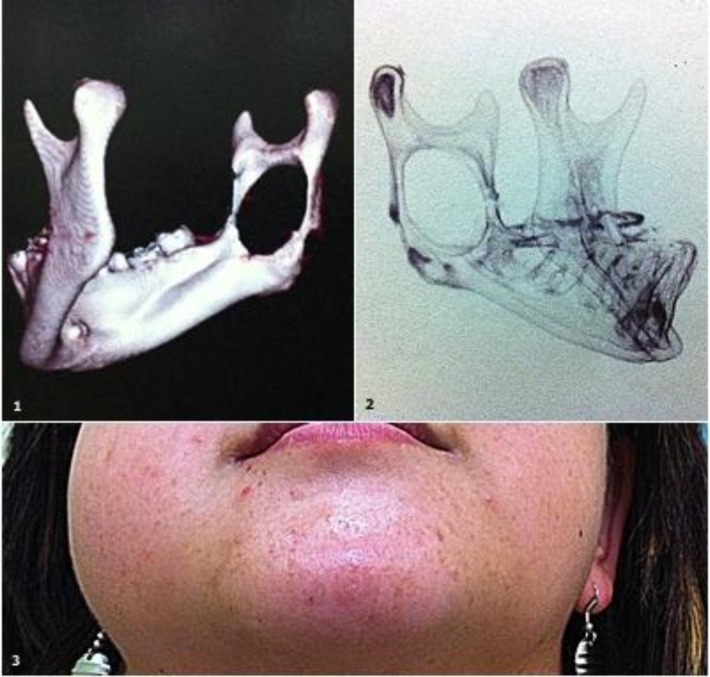
Illustration of Case 2.1: CT 3D reconstruction showing the substantial erosion in the right jaw. 2: Alternative view from the CT reconstruction. It is possible to note that the mandibular nerve is very close to the lesion. 3: Physical findings in the patient. Note the bulging in the right side of the jaw

A year after the initial surgery, a control CT scan showed persistence of the cystic area in the right ramus of the mandible approximately 7.5 cm in diameter, despite the evident thickening of the cortical basilar. Suspicion of a tumor recurrence was confirmed by subsequent histological analysis. Recently, the patient underwent resection of the residual lesion with removal of laminated white scaly tissue from the affected area, in addition to peripheral osteotomy reaching the bone with healthy appearance. There was no requirement for the use of reconstruction plates or any other kind of cavity fillers. The patient underwent a 6-month observational period without signs of recurrence (Fig.1).

***Case 3***

A male patient, 60 years of age, with a 20-year history of progressive bulging at the right angle of the mandible and chewing pain for approximately 1 year. He reported no bleeding, weight loss, or cervical masses. 

During a physical examination, we noticed a disfiguring angular bulging in the right mandibular, approximately 10×7 cm in size. This was indurated, painless, and jeopardizing the region of the mouth's floor and swelling the sulcus and the gum border. No neck lymphadenopathy was detected. 

A CT scan showed a cystic formation in the region of the angle and the right ramus of the mandible, approximately 8.0 cm wide, with irregular and inflated bone contours, in the presence of thin bone trabeculae, with evidence of cortical disruption, hypo-attenuating content without dental residues, and destroying the entire coronoid process.

**Fig2:**
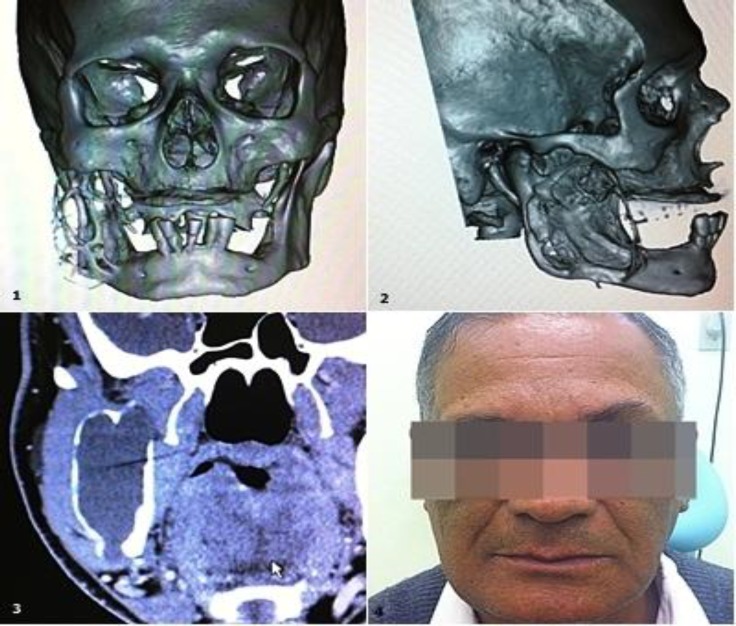
Illustration of Case1. 1: CT 3D reconstruction shows substantial erosion with irregular borders. 2: Alternative view from CT scan showing an approximately 8-cm lesion. 3: Coronal plan from CT showing thin bone trabeculae, evidence of cortical disruption, and hypo-attenuating content without dental residues and destroying the entire coronoid process. 4: Physical findings in the patient, showing bulging in the right mandibular of approximately 10×7 cm

The patient underwent tumor surgical resection via the intraoral route. We performed KOT confirmation in the frozen biopsy and in the final microscopy examination from paraffin. During surgery we observed substantial bone erosion, thin trabeculations, and numerous characteristic corneas structures. All the remaining bone from the ramus, angle, and coronoid process was removed by progressive osteotomy. The mandible was rebuilt with a 2.4 titanium plate, without bone grafts. We chose to sacrifice the inferior alveolar nerve involved by the tumor along its path. After 30 days, there was partial exposure of the plate portion within the oral cavity without evidence of infection or related complaints from the patient, who recovered the mandible contour, maintaining proper chewing. After a 9-month follow-up, no signs of recurrence were detected (Fig. 2).

## Discussion

As a lesion displaying more aggressive behavior when compared to other odontogenic cysts, the KOT presents, among other histopathological features, a layer of parakeratinized epithelium of lining which distinguishes it from other cysts with epithelial odontogenic cysts, called orthokera- tinized odontogenic cysts. With only partially elucidated histopathogenesis, these cysts are considered as separate entities. In order to better understand them, Aragaki evaluated the immunohistochemical profile from the keratin expression in each one (6). 

In a series of 32 surgically treated KOTs, Kuroyanagi observed significant expression of Ki-67 and p53 in the group with recurrences, suggesting that the evaluation of these marker proteins helps inform the decision regarding adjuvant procedures and that it has a prognostic value (7). According to Mendes, in the rapidly induced markers study, response to growth factors, tumor promoters, cytokines, bacterial endotoxins, oncogenes, hormones, and stress, such as COX-2, can increase our knowledge on the biological mechanisms involved in the development of these tumors (1).

Anticipating the proposition of future therapies, Zhang postulated that the development of synthetic active receptor antagonists or transcription factors from the Sonic hedgehog (SHH) signaling pathway may result in an effective treatment for this tumor. He suggested that inhibition of the Smoothened (SMO) by intracystic injection of a protein antagonist would be the treatment with the greatest potential (2).

For small KOTs, the most conservative procedure available (usually enucleation) would be mandatory. Endoscopic removal allows the careful exploration of difficult-to-reach areas through direct visualization, allowing not only the monitoring of the wall separation between the tumor and the inferior alveolar nerve, but also promoting the complete removal of the lesion (8). For larger tumors with bone cortical, coronoid process, or mandibular incision destruction, some authors suggest radical resection including transfacial access (9). In the cases described, we observed that the larger tumors were the result of years of negligence. The benign nature of these tumors is associated with the slow evolution and prospect of a possible recurrence, which leads to a more conservative attitude even in the presence of larger lesions. The presence of bone cortical erosion would be a negative prognostic factor. Although present in all our patients, we question whether it would be the product of more aggressive tumor or simply an expression of the longer evolution period.

In resections resulting in large residual fragility of the mandible, we recommend, as a preventive measure for possible secondary fracture of the bone, reinforcement with 2.4 titanium plates overlapping the lesion area, similar to the one from the reported case. With the expectation of substantial bone removal and interruption of the continuity, we suggest placing a plate before the tumor resection is finalized, since there is no requirement for en bloc removal. Therefore, a better quality mandibular reconstruction is accomplished by maintaining the correct positioning of the distal fragments associated with the condyle, particularly in the complete edentulous. The mandibular evaluation with computerized densitometry after the KOTs enucleation, showed progressive regeneration of the defect area without any grafting material, with a more significant increase in bone density 6 months after the surgery (10). Consistent with others authors (11), we believe that spontaneous bone regeneration occurs even in larger mandibular defects; and thus, in our patients, we do not use grafts or any kind of bone formation stimulating products. 

Owing to the frequent recurrence, a variety of adjuvant treatments have been proposed, including the removal of the peripheral bone (osteotomy), en bloc resection of the cyst with the surrounding bone, cryotherapy (freezing) with liquid azote, and use of Carnoy's fixative solution into the cavity after the enucleation. For Madras, a resection or enucleation supplemented with Carnoy's solution, with or without osteotomy, would result in a lower recurrence than with the enucleation or the marsupialization alone. However, the recurrence after marsupialization followed by enucleation did not differ from more aggressive therapies (12). In another study, Morgan noted that osteotomy with or without the use of Carnoy's solution showed a lower rate of recurrence compared with enucleation with or without the use of the same solution (13). In a retrospective analysis of a total of 68 patients, Zecha observed recurrence in 40% of the 10 marsupialized tumors with a follow-up of 58 months, and in 20.7% of the 58 enucleated with follow-up averaging 46 months (14). Because of the high recurrence rates, the author believes that further studies should investigate the possible benefits of supplementary treatments, especially the Carnoy's solution. Despite these reports, in a large systematic review of all literature related to the treatment of isolated KOT of the maxilla and the mandible, Sharif found no relevant published randomized controlled trials and states that no conclusion can be inferred regarding the effectiveness of these adjuvant interventions (3). The definition of the KOTs epithelial histological pattern and the expression of keratin raise questions about previous studies which sought to clarify the associated factors with these tumors recurrence; however, these works were probably associated with different types of lesions in their casuistry. In a retrospective study of 120 patients submitted to a simple enucleation and evaluated over a 10-year period, Pitak-Arnnop observed tumoral recurrence in 28 patients (26%), among which seven (6%) were multiple recurrences. However, the study did not identify a significant association with the radiological findings, the histological type, cortical perforation, or the lesion location (P>0.05) (15). In this study, the authors state that only 80 lesions showed parakeratosis, a fact that allows us to question whether the remaining cysts were in fact orthokeratinized, a lesion with a different evolution from the KOTs. In another study with patients treated for tumor decompression, August (16) observed the disappearance of the epithelium and interruption of the cytokeratin-10 production in 64% of cases; thus, questioning whether this change would be associated with lower recurrence rates. However, a recent study considers that the cytokeratin-10 expression would not occur in KOTs, but in orthokeratinized odontogenic cysts instead (6).

Boyne proposed an interesting thesis involving seven patients with multiple recurrences over a period up to 21 years. In this work, he highlights the histological analysis performed in resected hemimandibles from patients operated on several times, in which, besides the recurrence on the edges of old lesions, the presence of other lesions was also verified in different locations. From these observations, the author establishes the multifocal nature of the KOTs, and emphasizes that recurrences were not necessarily related to the surgeon's skills, but to the very nature of the lesion (17). We believe that further studies are needed to confirm these findings, but this behavior would undoubtedly be consistent with the current knowledge involving the biological behavior of KOTs.

Although extremely rare, it is important to remember that there are reports of squamous cell carcinomas derived from odontogenic keratocystic tumors (18).

## Conclusions

The benign nature of these tumors and their slow evolution justify conservative treatment, even in the case of large lesions. The knowledge relating to the biological profile of the KOTs is of great importance in order to better understand the evolution of these lesions; thus establishing more precise treatment and prognosis.
